# Editorial: Sustainable plant-based strategies for managing plant-parasitic nematodes

**DOI:** 10.3389/fpls.2026.1777643

**Published:** 2026-02-06

**Authors:** Mahfouz M.M. Abd-Elgawad, Claudia S.L. Vicente, Ashwell R. Ndhlala

**Affiliations:** 1 Plant Pathology Department, National Research Centre, Giza, Egypt; 2 Institute for Mediterranean Agriculture, Environment and Development, University of Évora, Évora, Portugal; 3 Department of Plant Production, Soil Science and Agric Eng., School of Agriculture and Environmental Sciences, University of Limpopo, Sovenga, South Africa

**Keywords:** grafting, nematicides, pests, plant extracts, root-knot nematodes, toxicity

Plant-parasitic nematodes (PPNs) are one of the most present pressing problems that cause considerable damage and yield losses on key crops, globally. Rapid and continuous world population growth, conflicts, and climate change are aggravating the related issues (Abd-Elgawad, 2025). Moreover, worldwide increasing restrictions on the use of chemical nematicides urge the need to develop and create safe and effective bionematicidal products and/or related devices. Therefore, current research on this subject is focused on understanding the modes of action of novel nematicidal approaches as well as to measure their effects on agriculture. Their end in view is to realize durable plant-based strategies for managing PPNs. The studies of how these inputs and accompanied techniques interact with other related biotic/abiotic factors are gaining momentum to optimize their functional roles in plant production and PPN control (Abd-Elgawad, 2022). Relevant efforts are always crowned by both avoiding biosecurity risks linked to PPNs and raising awareness of the stakeholders for best production practices.

This Research Topic hosts six original research articles and two reviews. The researchers span related findings of various crops with their associated production inputs and methods to combat PPNs and raise crop yield. A systematic review is designed to face PPN challenges and another one to maintain crop health against PPNs and ensure food supply chain resilience.

## Botanicals, organic amendments, and biocontrol agents for sustainable nematode control

There is increasing evidence that crop production practices can engage plant protection measures to improve crop yields and control pests/pathogens together (El-Nagdi et al., 2019; Abd-Elgawad et al., 2024). Vimala et al. confirmed that organic nutrient sources and biocontrol agents (BCAs) can severely affect the root-knot nematode (RKN) *Meloidogyne incognita*. The authors explored the impact of *Mentha* sp*icata* and *Piper longum* essential oils and extracts, along with *Bacillus amyloliquefaciens* and *B*. *subtilis* as well as organic amendments on *M*. *incognita* infecting tomato plants. While essential oils showed significant nematode-juvenile mortality and egg hatching inhibition, all treatments, comprising their combinations with organic amendments (farmyard manure, vermicompost, and paddy straw), could significantly reduce nematode populations, enhance tomato growth, and boost soil fertility. Such combinations performed comparably to the nematicide *Velum^®^ Prime* 400 SC. The results also showed a significant increase of soil organic carbon and NPK content (P < 0.05), demonstrating that these treatments could provide reliable, eco-friendly alternatives for *M*. *incognita* control, contributing to resilient/sustainable tomato production systems. Lillo et al. study evaluated the efficacy of six fertilization treatments, adjusted to provide equivalent units of N-fertilization, on *M*. *incognita*-infested greenhouse cucumber. Interestingly, the fresh chicken manure treatment yielded the highest cucumber production, despite insignificant differences in RKN-disease severity between treatments. Yet, the treatment was the most effective in minimizing RKN abundances in soil, followed by pelletized chicken manure, fresh cow manure, and finally composted cow manure. Contrary to organic amendments that enhanced the complexity of the soil food web, fast-release inorganic fertilizers led to its degradation and simplification. Inorganic fertilizers were the least effective in decreasing the nematode abundance. Notably, cucumber cultivation and fertigation throughout the crop cycle enriched the soil with nutrients, intensified the bacteria-dominated organic matter degradation channel, and further simplified the soil food web, in addition to suppressing RKN disease.

Könker et al. focused on the use of *Pochonia chlamydosporia* to synergistically reinforce systemic plant defense reaction of *Phacelia tanacetifolia* against *Meloidogyne hapla*. Transcriptome and metabolome analysis of plant leaves revealed that the metabolome was quite stable except for the first two days. Comparing *P*. *chlamydosporia* singly with *M*. *hapla* + *P*. *chlamydosporia* treatment manifested larger impacts after 6 compared to 2 days, aligning with the later root infestation by *P*. *chlamydosporia* compared to *M*. *hapla*. Shifts in transcripts and metabolites were higher in the combined treatment relative to the single inoculum which support the conclusion that *P*. *chlamydosporia* induces plant defense in a distinguished and beneficial manner when combined with *M*. *hapla*. Their results tentatively proposed that *P*. *chlamydosporia* application against *M*. *hapla* can be more effective via backing the underlying tritrophic interactions with specific additives, e.g. phytohormones or amino acids. This study provided valuable insights into the systemic response of plants to PPNs and a biocontrol fungus.

The study of Kisaakye et al. addressed chitin-enriched insect frass fertilizers as cheap and safe biorational alternatives for managing RKN (*M*. *incognita*). The authors tested seven chitin-fortified black soldier fly frass fertilizer extracts (chFE) for their suppressing of *M*. *incognita* and effects on spinach growth relative to a chemical nematicide, *Velum^®^ Prime*. Black solder fly (BSF) frass and BSF exuviae, merged in varying proportions to form seven compounds, were mixed with effective microorganisms, biochar and molasses, in a fermentation process to obtain chFE. *Meloidogyne incognita*-J2s paralysis reached 100% when exposed to either chFE or *Velum^®^ Prime*. Further, the J2 attained mortality using chFE (95%) was comparable to the value achieved using *Velum^®^ Prime* (96%). In all treatments, mortality increased with exposure time. Up to 85% suppression of nematode-gall development was achieved when spinach plants were grown in soil drenched with chFE. Suppressions of other RKN growth parameters were obtained. Also, chFE application significantly increased spinach root and shoot biomass considerably, compared to the commercial nematicide. These findings demonstrated the nematicidal potential of chFE and its benefits on crop production as a promising multipurpose, regenerative, and cost-effective input for PPN-sustainable management and boosting crop yield.

## Grafting and toxicological testing of phytonematicides for agro-sustainability

Grasping the role of grafting for PPN resistance in plants is essential in devising adequate management solutions. Following *M*. *incognita* infection, Li et al. found that the grafted progeny GHF1, F1 progeny of tobacco grafts ‘G278 (resistant rootstock) + Honghua Dajinyuan (HD, susceptible scion), showed notably enhanced resistance compared to its scion HD. This was characterized by raised chlorophyll levels, increased activity of phenylpropanoid metabolic enzymes and disease-related proteins, reduced membrane lipid peroxidation, and stable antioxidant enzyme levels. Among all combinations tested, GHF1 displayed the most robust resistance phenotype, stressing its potential as a superior germplasm resource. The study offers a sound framework for assessing the agronomic traits and stress responses of graft-derived tobacco progeny under PPN pressure by integrating phenotypic, physiological, and biochemical analyses.

Likewise, assessing the accumulation of phyto-nematicidal cytotoxicity is necessary for developing appropriate designs of safe and sustainable strategies. Ndhala et al. evaluated the toxicity of chemical residues of Nemarioc-AL and Nemafric-BL phytonematicides in tissues of tomato planted in different soil types. Cucurbitacins A and B were present in both phytonematicides. Accumulation of their residues in tomato tissues was independent of the treated soil type and type of phytonematicide applied to the soil. Such toxic compounds can cause severe illness in humans if ingested in large amounts, but the products can be used at lower concentrations to manage PPNs and avoid their toxicity. The authors stressed that farmers must use the non-toxic doses recommended for the safe use of both products, with negligible toxicity.

## Reviews of PPN challenges to bridge gaps for biosecurity and agro-sustainability

Biosecurity risks via introduction and spread of PPNs are exacerbated by increased global trade and climate change. Kantor et al. highlighted advanced biosecurity measures that could decrease PPN infestation rates by up to 70%. Managing PPN-related biosecurity risks should address PPN-modes of transmission, factors increasing their risk of infestation, and their impact on food safety/security. Also, modern molecular diagnostics of PPNs, integrated plant management plans, and emerging geospatial surveillance and analysis systems (spectral imaging, change-detection analysis) should be manipulated to enhance crop health and secure the food supply. Moreover, Elhady et al. recommended proper assembly, diversity, and a unified and purpose-driven framework to face PPN problems in the arid regions. The authors proposed integrating beneficial microbial-based products and soil development practices in hygienic farming as resilient and sustainable solutions for PPN management. Detrimental agriculture practices were specified to be avoided.
